# The influence of C-reactive protein-triglyceride-glucose index (CTI) on the prognosis of heart failure patients with different ejection fractions

**DOI:** 10.3389/fcvm.2026.1808481

**Published:** 2026-06-08

**Authors:** Xinuo Ma, Feng Wang, Dajin Liu, Ying Li, Ping Xia, Yunyun Xu, Han Xia, Yujuan Peng, Lixing Chen

**Affiliations:** 1 Department of Cardiology, First Affiliated Hospital of Kunming Medical University, Kunming, China; 2 Department of Pathogenic Biology and Immunology, Kunming Medical University, Kunming, China; 3 Medical Records and Statistics Department, First Affiliated Hospital of Kunming Medical University, Kunming, China; 4 Internal Medicine Department for Officials, First Affiliated Hospital of Kunming Medical University, Kunming, China

**Keywords:** C-reactive protein-triglyceride-glucose index, heart failure, inflammation, insulin resistance, metabolism

## Abstract

**Objective:**

The C-reactive protein-triglyceride-glucose index (CTI) is a novel biomarker integrating inflammation and insulin resistance. Its prognostic value for all-cause mortality in patients with chronic heart failure (CHF) across the ejection fraction (EF) spectrum remains unclear.

**Methods:**

Patients were categorized into low- and high-CTI groups based on a restricted cubic spline-derived cutoff. Kaplan–Meier curves were used to compare survival, and Cox proportional hazards models were used to assess the association between CTI and all-cause mortality. Receiver operating characteristic (ROC) analysis and subgroup analyses were performed.

**Results:**

A total of 1,130 patients with CHF were included. Kaplan–Meier survival analysis showed that patients with elevated CTI levels had a significantly higher risk of all-cause mortality compared to those with lower CTI levels. Multivariable-adjusted Cox proportional hazards analysis further confirmed that CTI levels were independently associated with increased all-cause mortality. Subgroup analysis demonstrated that elevated CTI levels were associated with higher all-cause mortality in patients with heart failure with reduced ejection fraction (HFrEF) and mildly reduced ejection fraction (HFmrEF).

**Conclusions:**

CTI is an independent prognostic marker for all-cause mortality in patients with CHF across different EF subtypes and may serve as a useful risk stratification biomarker.

## Introduction

Heart failure (HF) is a clinical syndrome that exhibits significant heterogeneity. This terminal stage of cardiovascular diseases has emerged as a significant public health challenge in China and on a global scale, attributable to its high incidence and mortality rates. According to the Global Burden of Disease (GBD) study, the global prevalence of heart failure more than doubled from 25.43 million cases in 1990 to 55.5 million cases in 2021 (1). Concurrently, China has also witnessed a substantial surge in heart failure cases, with estimates indicating a 208.4% increase compared to three decades prior, reaching 14.3 million cases in 2023 (2). The aging of the population and the increase in risk factors have led to a concomitant rise in the incidence of heart failure on an annual basis. This increase in incidence is becoming increasingly evident among young people, accompanied by a shift towards heart failure that preserves ejection fraction. Furthermore, heart failure imposes a substantial medical burden. A population study conducted in various Chinese cities in 2017 demonstrated that both the prevalence and incidence of heart failure increased with age. The mean cost of hospitalization per individual was $4,406.8, and the proportion of hospitalizations of at least three instances was 93.0% (3). Consequently, it is imperative to identify and diagnose individuals at high risk of heart failure as expeditiously as possible.

The 2021 European Society of Cardiology Heart Failure Guidelines propose a classification system for heart failure (HF) based on left ventricular ejection fraction (LVEF). The classification system delineates three phenotypes: heart failure with reduced ejection fraction (HFrEF, LVEF ≤40%), heart failure with mildly reduced ejection fraction (HFmrEF, LVEF 41%–49%), and heart failure with preserved ejection fraction (HFpEF, LVEF ≥50%) (4).

Inflammation has been identified as a pivotal factor in the development and progression of cardiac hypertrophy. Several inflammatory markers, including C-reactive protein (CRP), have been demonstrated to be significantly associated with the progression of heart failure (HF) (5). Insulin resistance is a hallmark of heart failure. Multiple mechanisms—such as fatty toxicity, sympathetic overactivation, inflammation, oxidative stress, and fibrosis—can cause myocardial damage and eventually lead to ventricular remodeling. Higher insulin resistance in youth is a risk factor for poor left ventricular remodeling and left ventricular dysfunction in later life. The TyG index is regarded as a reliable indicator for evaluating insulin resistance at present. The C-reactive protein-triglyceride-glucose index (CTI) is an innovative marker that combines C-reactive protein (CRP) and triglyceride-glucose (TyG) indices, enabling simultaneous assessment of inflammatory status and the severity of insulin resistance. Preliminary research has demonstrated that CTI functions as an autonomous predictor of stroke, depression, and cardiovascular diseases. However, there is a paucity of further research on the prognosis of different types of heart failure. The objective of this study is to assess the impact of CTI on the clinical prognosis of patients with heart failure and varying ejection fractions.

## Materials and methods

### Study population

The present study retrieved a total of 1,221 medical records of patients diagnosed with chronic heart failure at the First Affiliated Hospital of Kunming Medical University from January 2017 to October 2021. The inclusion criteria encompassed patients diagnosed with congestive heart failure (CHF) classified as grade III or IV according to the New York Heart Association (NYHA) functional classification, in addition to those exhibiting brain natriuretic peptide (BNP) levels ≥500 pg/mL. The exclusion criterion was the lack of necessary data (such as routine blood tests or cardiac ultrasound data). Combined with other serious diseases (such as malignant tumors, infectious diseases, blood diseases, or severe renal or liver dysfunction) or a lack of follow-up data. Finally, 1,130 patients were included in this study. The diagnosis of CHF was based on the 2021 ESC Guidelines for the Management of Acute and Chronic Heart Failure.

### Statistical analysis

For each enrolled CHF patient, baseline data were obtained through medical records, including: (1) demographic and physical measurement data [age, gender, body mass index (BMI), systolic blood pressure (SBP), diastolic blood pressure (DBP)]; (2) Lifestyle characteristics, clinical history and current medication status (smoking status, drinking status, history of hypertension, history of diabetes, history of stroke, etc.); (3) Laboratory test data (White blood cells (WBC), red blood cells (RBC), platelets (PLT), absolute neutrophil count (Neu), lymphocytes (LYM), monocytes (MONO), B-type natriuretic peptide (BNP), sodium, potassium, chlorine, albumin (Alb), uric acid (UA), glomerular filtration rate (GFR), alanine aminotransferase (AST), alanine aminotransferase (ALT), triglycerides (TG), fasting blood glucose (FBG), C-reactive protein (CRP), etc.); (4) Electrocardiogram and echocardiogram data;

Survival data were collected through telephone interviews with patients or their families. All-cause mortality was defined as the endpoint of the study.

### Calculation of CTI

CRP-TyG Index (CTI) consists of CRP and TyG. The CTI index is calculated based on the established formula: CTI = 0.412 ×Ln [CRP (mg/L)] + Ln [TG (mg/dl) ×FPG (mg/dl)/2]

### Statistical analysis

The clinical role of CTI was predefined as a risk stratification tool for prognostic assessment in different HF types. The patients were divided into two groups, low CTI (CTI ≤ 1.98, *n* = 565) and high CTI (CTI > 1.98, *n* = 565), based on the inflection point identified from the restricted cubic spline plot. Continuous data with a normal distribution were presented as mean ± standard deviation (SD); otherwise, they were presented as median (interquartile range). Categorical data were expressed as counts and percentages. To compare patient groups with different CTI levels, the independent sample t-test was used for normally distributed continuous data, the Mann–Whitney U test for non-normally distributed continuous data, and the chi-square test for categorical data. To further explore the impact of CTI on clinical prognosis across different HF ejection fraction subtypes, the entire cohort was divided based on left ventricular ejection fraction (LVEF) into the HFrEF plus HFmrEF group (LVEF < 50%, *n* = 688) and the HFpEF group (LVEF ≥ 50%, *n* = 442).

Survival curves were plotted using the Kaplan–Meier method and compared using the log-rank test. The multivariate Cox proportional hazards model was applied to estimate the hazard ratio (HR) for the association between CTI and all-cause mortality. Variables for multivariable Cox regression were selected based on clinical relevance from the literature and univariate analysis with a threshold of *p* < 0.05. Although diabetes mellitus was significantly associated with the outcome in univariate analysis (*p* < 0.001), it was not included in the primary multivariable models. A *post-hoc* sensitivity analysis adding diabetes to the models was conducted to evaluate its impact on the hazard ratio of CTI. To assess potential overfitting, internal validation using 500 bootstrap resamples was performed for the HFpEF subgroup (the smallest sample size among the three groups), and the optimism-corrected C-index was calculated. Receiver operating characteristic (ROC) analysis was performed based on three models to evaluate the discriminative ability of CTI for distinguishing patients at higher risk of death. To directly compare CTI with its individual components (CRP and TyG), three separate Cox models were constructed based on the original Model 2 (adjusted for Age, sex, lgBNP, RBC, Sodium, and GFR) by adding CRP, TyG, or CTI, respectively. Model performance was compared using the likelihood ratio test, AIC, BIC, and C-index. Subgroup analysis was conducted to assess whether the prognostic association of CTI with all-cause mortality varied across different patient subgroups. The clinical role of CTI was defined as risk stratification rather than diagnostic or therapeutic monitoring. Based on the RCS-derived threshold, patients were categorized into low- and high-CTI groups to evaluate their prognostic value across HF subtypes.

Data were statistically analysed using SPSS 31.0 and R 4.4.0. A bilateral *p*-value < 0.05 was considered statistically significant.

## Result

### Baseline patient characteristics

**Figure 1:**
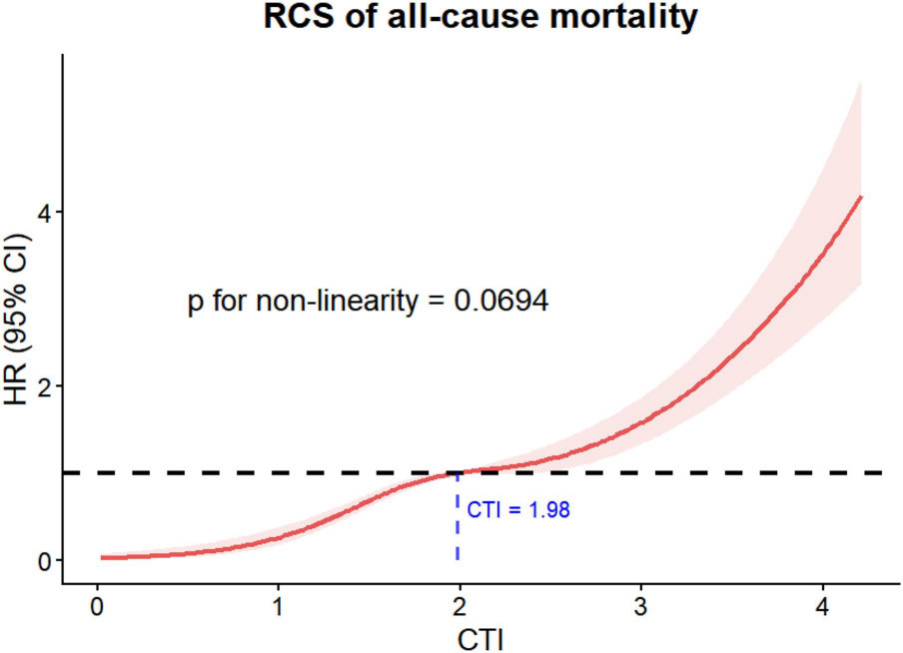
Restricted cubic bar graphs based on optimal thresholds for CTI in patients with different subtypes of CHF.

In this study, 1,130 patients with heart failure were enrolled (mean age: 66.87 ± 12.46 years; 38.10% female). The median follow-up duration for the entire cohort was 754.5 days (IQR: 343.25–1124.5). During this period, a total of 530 deaths (46.9%) occurred. The mortality rate was 49.3% (339/688) in the HFrEF + HFmrEF group and 43.2% (191/442) in the HFpEF group. A restricted cubic spline plot was generated to identify an inflection point at 1.98 (*p* < 0.05), which divided patients into two groups (Figure 1). Baseline characteristics of the low CTI (*n* = 565) and high CTI (*n* = 565) groups are presented in Table 1. Compared with the low CTI group, the high CTI group had lower levels of red blood cells (RBC), lymphocytes (LYM), serum sodium, serum chloride, albumin (ALB), and glomerular filtration rate (GFR) (all *p* < 0.05). Conversely, the high CTI group had significantly higher levels of log-transformed B-type natriuretic peptide (lgBNP), white blood cells (WBC), neutrophils (Neu), monocytes (MONO), C-reactive protein (CRP), aspartate aminotransferase (AST), creatinine, uric acid (UA), fasting blood glucose (FBG), triglycerides (TG), and fibrinogen (Fib) (all *p* < 0.05). Regarding medication history, the proportion of patients using SGLT-2 inhibitors, beta-blockers, diuretics, and ACEI/ARB/ARNI (angiotensin-converting enzyme inhibitors/angiotensin receptor blockers/angiotensin receptor neprilysin inhibitors) was significantly higher in the high CTI group. Additionally, the incidence of atrial fibrillation was lower in the high CTI group (all *p* < 0.05).

**Table 1:** Baseline characteristics according to the CTI.

Characteristics	Total (*n* = 1,130)	low-CTI (*n* = 565)	high-CTI (*n* = 565)	*p* value
Basic characteristics				
Age (years)	66.87 ± 12.46	65.57 ± 12.90	68.17 ± 11.88	p＜0.001
Female, *n* (%)	430 (38.10%)	219 (38.80%)	211 (37.30%)	p＜0.001
HR (bpm)	85.14 ± 21.09	82.51 ± 20.24	87.76 ± 21.62	p＜0.001
Blood pressure (mmHg)	122.11/76.25	120.98/75.93	123.25/76.56	0.05/0.244
BMI (kg/m^2^)	23.00 ± 3.85	22.68 ± 3.83	23.32 ± 3.85	p＜0.05
NYHA class Ⅳ, *n* (%)	422 (37.30%)	189 (33.50%)	233 (41.20%)	p＜0.001
Medical history, *n* (%)				
Coronary artery disease	582 (51.50%)	255 (45.10%)	327 (57.90%)	0.312
Hypertension	628 (55.60%)	284 (50.30%)	344 (60.90%)	p＜0.001
Diabetes	317 (28.10%)	90 (15.90%)	227 (40.20%)	p＜0.001
Atrial fibrillation	380 (33.60%)	206 (36.50%)	174 (30.80%)	p＜0.001
Stroke	154 (13.60%)	59 (10.40%)	95 (16.80%)	p＜0.001
Treatment				
SGLT-2I	247 (21.90%)	113 (20.00%)	134 (23.70%)	p＜0.001
*β*-receptor blockers	792 (70.1%)	389 (68.8%)	403 (71.3%)	p＜0.001
Diuretics	896 (79.3%)	446 (78.9%)	450 (79.6%)	p＜0.001
ACEI/ARB/ARNI	639 (56.5%)	305 (54.0%)	334 (59.1%)	p＜0.001
CRT/CRTD	109 (9.60%)	51 (9.00%)	58 (10.30%)	p＜0.001
Laboratory indicators				
lgBNP	3.17 ± 0.28	3.14 ± 0.27	3.19 ± 0.29	p＜0.05
WBC (10^9/L)	6.94 (5.56,9.05)	6.43 (5.30,8.13)	7.40 (5.94,10.09)	p＜0.001
RBC (10^12/L)	4.55 ± 0.76	4.59 ± 0.72	4.51 ± 0.80	p＜0.05
PLT (10^9/L)	201.36 ± 78.66	198.61 ± 75.37	204.11 ± 81.78	0.120
Neu (10^9/L)	4.54 (3.51,6.50)	4.21 (3.30,5.56)	5.12 (3.82,7.43)	p＜0.001
LYM (10^9/L)	1.38 (1.02,1.83)	1.45 (1.08,1.86)	1.31 (0.99,1.77)	p＜0.05
MONO (10^9/L)	0.54 (0.40,0.72)	0.52 (0.39,0.69)	0.57 (0.42,0.75)	p＜0.001
CRP (mg/L)	7.50 (3.00,21.38)	3.35 (1.88,7.59)	18.46 (7.31,43.60)	p＜0.001
Sodium (mmol/L)	141.00 ± 4.45	141.60 ± 4.47	140.39 ± 4.35	p＜0.001
Potassium (mmol/L)	3.94 ± 0.61	3.94 ± 0.57	3.95 ± 0.65	0.379
Chlorine (mmol/L)	102.91 ± 4.71	103.64 ± 4.65	102.17 ± 4.67	p＜0.001
Alb (g/L)	36.71 ± 4.53	37.40 ± 4.27	36.03 ± 4.68	p＜0.001
ALT (IU/L)	25.30 (16.78,42.65)	24.30 (16.70,39.55)	27.20 (16.80,48.70)	0.056
AST (IU/L)	28.65 (20.00,43.53)	27.00 (20.00,40.00)	30.00 (20.55,48.95)	p＜0.05
TB (umol/L)	15.90 (10.43,24.70)	16.35 (10.90,25.38)	15.40 (9.90,23.50)	0.061
Creatinine (umol/L)	103.85 (83.57,134.53)	99.00 (81.75,124.80)	109.20 (85.15,142.60)	p＜0.001
UA (umol/L)	478.00 (371.50,589.50)	466.00 (361.25,571.35)	491.60 (393.55,607.75)	p＜0.05
FBG (mmol/L)	5.02 (4.16,6.40)	4.56 (4.03,5.20)	5.62 (4.60,8.08)	p＜0.001
GFR (mL/min)	43.70 (32.28,56.37)	45.97 (35.53,58.03)	41.82 (29.32,54.63)	p＜0.001
TC (mmol/L)	3.59 (2.96,4.25)	3.56 (2.97,4.18)	3.59 (2.94,4.31)	0.598
TG (mmol/L)	1.11 (0.87,1.48)	0.97 (0.80,1.21)	1.32 (1.02,1.77)	p＜0.001
LDL (mmol/L)	2.21 (1.66,2.82)	2.19 (1.64,2.74)	2.27 (1.69,2.98)	0.076
Fib (g/L)	3.37 (2.71,4.13)	3.13 (2.60,3.81)	3.67 (2.89,4.69)	p＜0.001
TT (sec.)	18.20 (17.00,19.50)	18.40 (17.30,19.50)	18.10 (16.90,19.60)	0.063
APTT (sec.)	43.16 ± 12.24	42.58 ± 9.51	43.74 ± 14.44	0.056
ECG parameters and cardiac ultrasound index				
QRSd (ms)	115.47 ± 30.00	116.29 ± 30.33	114.65 ± 29.67	0.180
AOR (mm)	27.94 ± 4.37	27.87 ± 4.60	28.02 ± 4.12	0.287
Lad (mm)	42.37 ± 9.30	42.93 ± 9.32	41.81 ± 9.26	p＜0.05
LVd (mm)	56.30 ± 12.62	57.40 ± 13.21	55.21 ± 11.92	p＜0.05
Rad (mm)	51.91 ± 12.21	53.34 ± 12.75	50.47 ± 11.48	p＜0.001
RVd (mm)	67.24 ± 16.62	67.74 ± 16.81	66.73 ± 16.42	0.057

SBP, systolic blood pressure; DBP, diastolic blood pressure; BMI, body mass index; NYHA, New York Heart Association; SGLT2i, sodium-glucose cotransporter 2 inhibitor; ACEI, angiotensin-converting enzyme inhibitors; ARB, angiotensin receptor blocker; ARNI, angiotensin receptor neprilysin inhibitor; CRT, cardiac resynchronization therapy; CRTD, cardiac resynchronization therapy- cardioverter -defibrillator; BNP, B-type natriuretic peptide; WBC, white blood cell; RBC, red blood cell; Neu, neutrophile; LYM, lymphocyte; NLR, neutrophil-to-lymphocyte ratio; CRP, C-reactive protein; Hb, hemoglobin; PLT platelets; ALB, albumin; ALT, aspartate transaminase; AST, alanine aminotransferase; UA, uric acid; TC:total cholesterol; GFR, glomerular filtration rate; FBG, fasting blood-glucose; TB, total bilirubin; HDL, high-density lipoproteins; LDL, low-density lipoproteins; Lad, Left atrial diameter; LVd, left ventricular diameter; Rad, right atrial diameter; RVd, right ventricular diameter.

### CTI and all-cause mortality

A restricted cubic spline plot was generated based on the association between CTI and all-cause mortality (Figure 1). The findings suggested a near non-linear association between CTI and the risk of all-cause mortality (non-linear *p* = 0.0694). Specifically, when using a CTI value of 1.98 as the reference point (HR = 1.0), the hazard ratio (HR) for all-cause mortality remained close to 1.0 when CTI values were below this threshold. Conversely, CTI values above this threshold were associated with a significant increase in all-cause mortality risk.

**Figure 2:**
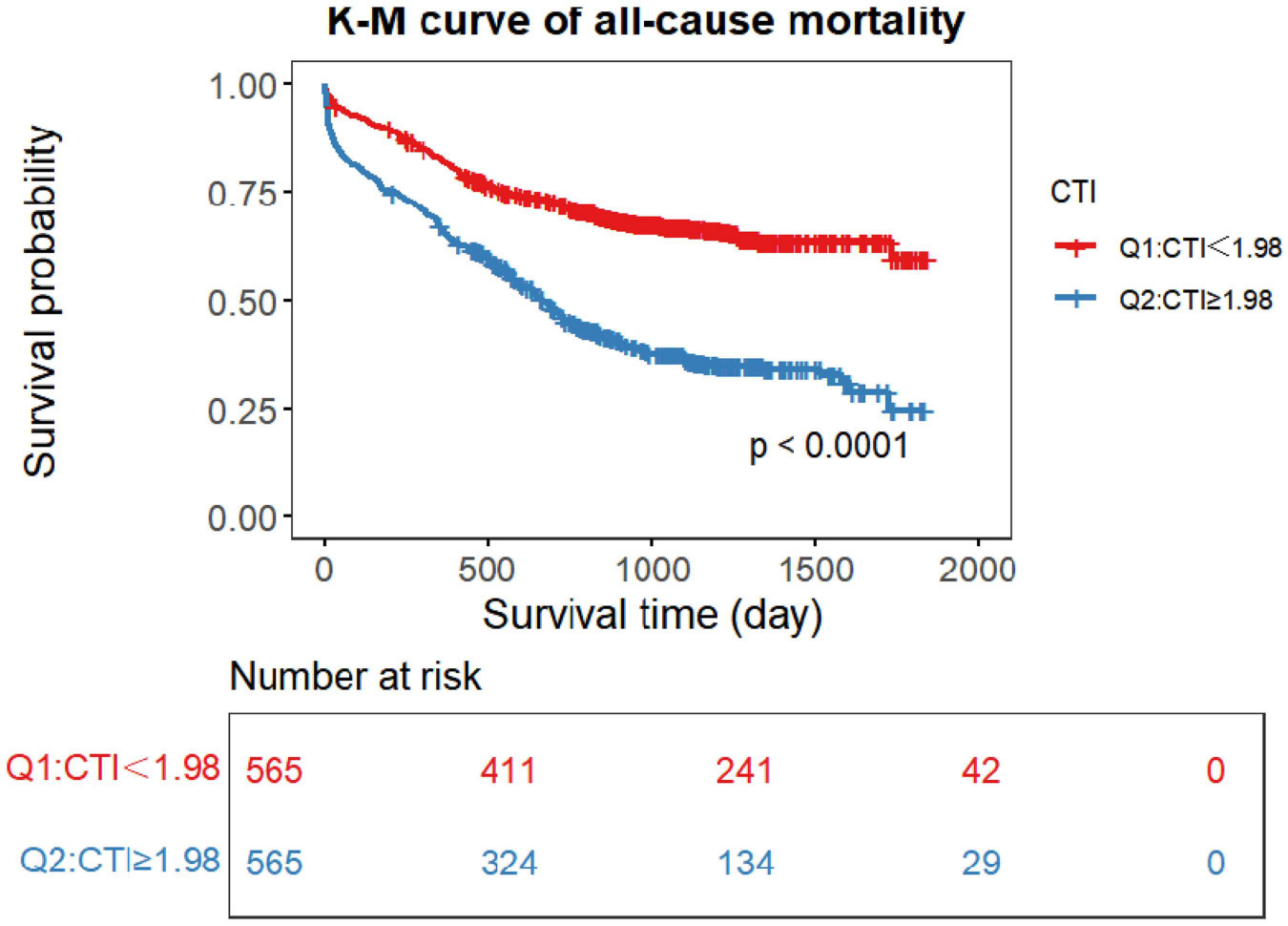
Kaplan–meier survival curves of all patients with chronic heart failure at different CTI level groups.

**Figure 3:**
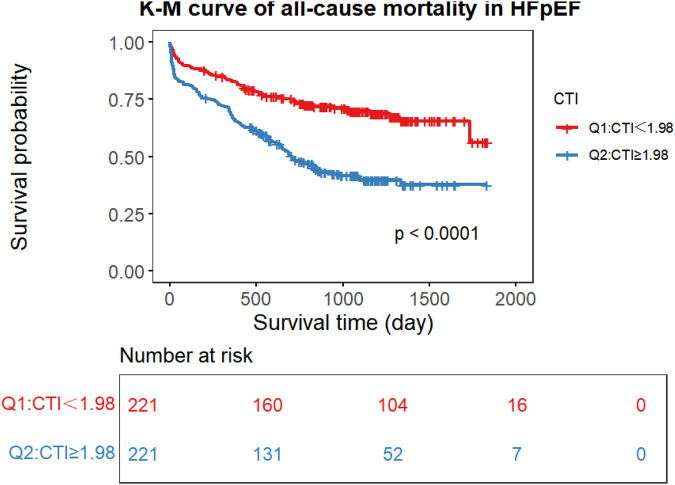
Kaplan–meier survival curves of HFpEF patients in different CTI level groups.

**Figure 4:**
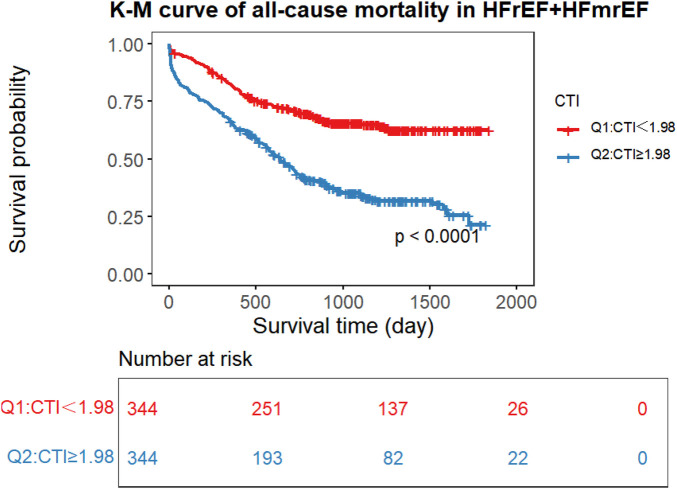
Kaplan–meier survival curves of HFrEF plus HFmrEF patients in different CTI level groups.

To further investigate the relationship between CTI and all-cause mortality in heart failure patients with different ejection fractions, we plotted Kaplan–Meier curves (Figures 2, 3, 4). The results showed that patients with high CTI levels had a significantly higher risk of all-cause mortality compared to those with low CTI levels (log-rank *p* < 0.001). This elevated risk was consistently observed across all subgroups: patients with heart failure with preserved ejection fraction (HFpEF), those with reduced and mildly reduced ejection fraction (HFrEF + HFmrEF) (HFrEF + HFmrEF) (*p* < 0.001).

### CTI as an independent predictor

In all patients with chronic heart failure (CHF), univariate Cox proportional hazards analysis showed that CTI was significantly associated with all-cause mortality (HR = 2.101, 95% CI: 1.908–2.313,*p* < 0.001). After adjusting for age, sex, BMI, NYHA functional class, LgBNP, white WBC, Neu, LYM, RBC, serum sodium, chloride, ALB, AST, ALT, urea, and GFR and Fib, multivariable Cox proportional hazards regression analysis showed that CTI remained significantly associated with all-cause mortality (HR 1.697,95% CI 1.522–1.891,*p* < 0.001)(Table 2).

**Table 2:** Univariable and multivariable analysis of Cox proportional hazards models for all the patients with CHF.

All patients with CHF				
	Univariable		Multivariable	
	HR (95% CI)	*p*	HR (95% CI)	*p*
Age	1.030 (1.022,1.038)	＜0.001	1.011 (1.002,1.020)	＜0.05
Sex (ref:Female)	1.050 (0.880,1.252)	0.590	1.043 (0.865,1.258)	0.657
BMI	0.948 (0.926,0.970)	＜0.001	0.966 (0.942,0.990)	＜0.05
NYHA (ref:Ⅲ)	2.403 (2.026,2.850)	＜0.001	1.800 (1.503,2.155)	＜0.001
Smoking (ref:no)	1.105 (0.926,1.319)	0.268		
Hypertension	1.027 (0.865,1.219)	0.762		
lgBNP	5.337 (3.889,7.324)	＜0.001	2.560 (1.820,3.602)	＜0.001
WBC	1.062 (1.041,1.082)	＜0.001	0.960 (0.916,1.007)	0.093
Neu	1.091 (1.068,1.114)	＜0.001	1.068 (1.013,1.126)	＜0.05
LYM	0.720 (0.626,0.828)	＜0.001	1.018 (0.883,1.173)	0.810
RBC	0.732 (0.651,0.823)	＜0.001	0.892 (0.786,1.012)	0.075
Sodium	0.935 (0.917,0.953)	＜0.001	1.000 (0.977,1.024)	0.999
Chlorine	0.930 (0.913,0.947)	＜0.001	0.962 (0.940,0.984)	＜0.001
ALB	0.924 (0.906,0.942)	＜0.001	0.947 (0.926,0.968)	＜0.001
ALT	1.003 (1.002,1.004)	＜0.001	0.999 (0.997,1.001)	0.414
AST	1.005 (1.004,1.006)	＜0.001	1.004 (1.001,1.006)	＜0.05
Urea	1.057 (1.043,1.070)	＜0.001	0.996 (0.978,1.013)	0.622
GFR	0.975 (0.970,0.980)	＜0.001	0.993 (0.986,0.995)	＜0.05
Fib	1.097 (1.026,1.172)	0.007	0.943 (0.878,1.011)	0.100
TG	1.002 (0.875,1.146)	0.982		
FBG	1.078 (1.056,1.101)	＜0.001		
CRP	1.013 (1.011,1.014)	＜0.001		
CTI	2.101 (1.908,2.313)	＜0.001	1.697(1.522,1.891)	＜0.001

BMI, body mass index; NYHA, New York Heart Association; BNP, B-type natriuretic peptide; WBC, white blood cell; Neu, neutrophil; LYM, lymphocyte; Hb, hemoglobin; RBC, red blood cell; ALB, albumin; ALT, aspartate transaminase; AST, alanine aminotransferase; GFR, glomerular filtration rate; FBG, fasting blood-glucose; UA, uric acid; TC, total cholesterol; CRP, C-reactive protein; Fib, fibrinogen; TC, Triglycerides.

In the HFpEF subgroup, CTI showed a stronger association (HR = 1.906, 95%CI: 1.565–2.321, *p* < 0.001), whereas in the HFrEF + HFmrEF subgroup, the association was attenuated but remained significant (HR: 1.592, 95%CI: 1.389–1.824, *p* < 0.001) (Table 3).

**Table 3:** Univariable and multivariable analysis of Cox proportional hazards models for the patients with HFrEF and HFpEF plus HFmrEF.

	HFpEF				HFrEF + HFmrEF			
	Univariable		Multivariable		Univariable		Multivariable	
	HR (95% CI)	*p*	HR (95% CI)	*p*	HR (95% CI)	*p*	HR (95% CI)	*p*
Age	1.044 (1.029,1.058)	＜0.001	1.036 (1.020,1.054)	＜0.001	1.026 (1.016,1.035)	＜0.001	1.007 (0.996,1.019)	0.195
Gender (ref:Female)	1.108 (0.831,1.477)	0.486	1.019 (0.739,1.404)	0.910	0.992 (0.793,1.241)	0.943	1.005 (0.787,1.283)	0.971
BMI	0.951 (0.916,0.987)	＜0.05	0.938 (0.897,0.981)	＜0.05	0.946 (0.918,0.975)	＜0.001	0.958 (0.926,0.990)	＜0.05
NYHA (ref:Ⅲ)	2.616 (1.968,3.478)	＜0.001	2.565 (1.865,3.527)	＜0.001	2.270 (1.833,2.811)	＜0.001	1.670 (1.318,2.115)	＜0.001
Smoking	1.197 (0.884,1.621)	0.245			1.042 (0.837,1.297)	0.714		
Hypertension	0.879 (0.656,1.177)	0.387			1.152 (0.931,1.426)	0.193		
lgBNP	6.552 (3.920,10.952)	＜0.001	2.715 (1.505,4.898)	＜0.001	6.112 (3.923,9.521)	＜0.001	2.898 (1.766,4.757)	＜0.001
WBC	1.050 (1.017,1.085)	＜0.05	0.971 (0.882,1.068)	0.540	1.070 (1.045,1.097)	＜0.001	0.969 (0.918,1.022)	0.245
Neu	1.074 (1.038,1.112)	＜0.001	1.009 (0.911,1.119)	0.859	1.104 (1.075,1.134)	＜0.001	1.070 (1.006,1.137)	＜0.05
LYM	0.770 (0.624,0.950)	＜0.05	1.039 (0.830,1.302)	0.738	0.684 (0.568,0.824)	＜0.001	0.986 (0.811,1.198)	0.888
RBC	0.673 (0.556,0.815)	＜0.001	0.838 (0.677,1.036)	0.103	0.760 (0.654,0.883)	＜0.001	0.919 (0.780,1.084)	0.318
Sodium	0.942 (0.912,0.973)	＜0.001	1.026 (0.990,1.064)	0.156	0.931 (0.908,0.954)	＜0.001	0.989 (0.957,1.022)	0.499
Chlorine	0.923 (0.898,0.949)	＜0.001	0.935 (0.902,0.968)	＜0.001	0.935 (0.913,0.958)	＜0.001	0.978 (0.947,1.010)	0.175
ALB	0.917 (0.889,0.946)	＜0.001	0.957 (0.922,0.992)	＜0.05	0.925 (0.901,0.949)	＜0.001	0.941 (0.913,0.969)	＜0.001
ALT	1.005 (1.003,1.007)	＜0.05	0.997 (0.993,1.002)	0.219	1.003 (1.002,1.004)	＜0.001	1.000 (0.998,1.003)	0.855
AST	1.007 (1.005,1.009)	＜0.001	1.008 (1.003,1.012)	＜0.05	1.004 (1.003,1.006)	＜0.001	1.001 (0.998,1.004)	0.428
Urea	1.056 (1.033,1.079)	＜0.001	1.028 (0.995,1.062)	0.100	1.056 (1.040,1.074)	＜0.001	0.985 (0.961,1.009)	0.218
GFR	0.976 (0.967,0.984)	＜0.001	1.004 (0.994,1.014)	0.431	0.973 (0.967,0.980)	＜0.001	0.993 (0.984,1.002)	0.132
FBG	1.092 (1.060,1.125)	＜0.001			1.067 (1.037,1.098)	＜0.001		
UA	1.001 (1.000,1.002)	＜0.05	1.000 (0.999,1.001)	0.746	1.002 (1.001,1.002)	＜0.001	1.001 (1.000,1.002)	＜0.05
TG	1.082 (0.907,1.292)	0.380			0.937 (0.782,1.123)	0.479		
CRP	1.013 (1.010,1.015)	＜0.001			1.012 (1.010,1.014)	＜0.001		
CTI	2.126 (1.837,2.461)	＜0.001	1.906(1.565,2.321)	＜0.001	2.074(1.824,2.358)	＜0.001	1.592(1.389,1.824)	＜0.001

BMI, body mass index; NYHA, New York Heart Association; BNP, B-type natriuretic peptide; WBC, white blood cell; Neu, neutrophil; LYM, lymphocyte; Hb, hemoglobin; RBC, red blood cell; ALB, albumin; ALT, aspartate transaminase; AST, alanine aminotransferase; GFR, glomerular filtration rate; FBG, fasting blood-glucose; UA, uric acid; TC, total cholesterol; CRP, C-reactive protein; Fib, fibrinogen; TC, Triglycerides.

Sensitivity analysis adding diabetes mellitus as an additional covariate yielded hazard ratios for CTI that were nearly identical to those from the primary models (Supplementary Table 2). For the overall cohort, the HR changed from 1.697 (95% CI: 1.522–1.891) to 1.740 (95% CI: 1.552–1.950). For the HFrEF + HFmrEF subgroup, the HR changed from 1.592 (95% CI: 1.389–1.824) to 1.624 (95% CI: 1.407–1.874). For the HFpEF subgroup, the HR changed from 1.906 (95% CI: 1.565–2.321) to 1.967 (95% CI: 1.592–2.431).

### Overfitting risk assessment

The events-per-variable (EPV) ratios were 29.4 for the overall cohort (530 events/18 covariates), 18.8 for the HFrEF plus HFmrEF subgroup (339 events/18 covariates), and 10.6 for the HFpEF subgroup (191 events/18 covariates). For the HFpEF subgroup (the model with the lowest EPV), bootstrap internal validation with 500 resamples yielded an apparent C-index of 0.800 and a bias-corrected C-index of 0.809 (95% CI: 0.774–0.838), with an optimism of −0.008, indicating no evidence of overfitting. The hazard ratio for CTI remained stable after bootstrap correction (original HR: 1.906, 95% CI: 1.565–2.321; bias-corrected HR: 1.952, 95% CI: 1.524–2.439). Detailed results are presented in Supplementary Table 3.

### Predictive ability of the CTI

To further evaluate the discriminative ability of CTI for identifying patients at higher risk of all-cause mortality in heart failure patients, we constructed three models and performed ROC curve analysis. Of note, these AUC estimates reflect within-cohort discrimination and do not imply external predictive performance. Model 1 adjusted for age and sex. Model 2 further adjusted for lgBNP, red blood cell count, serum sodium, and glomerular filtration rate (GFR) based on Model 1. Model 3 was further adjusted for CTI based on Model 2.

**Figure 5:**
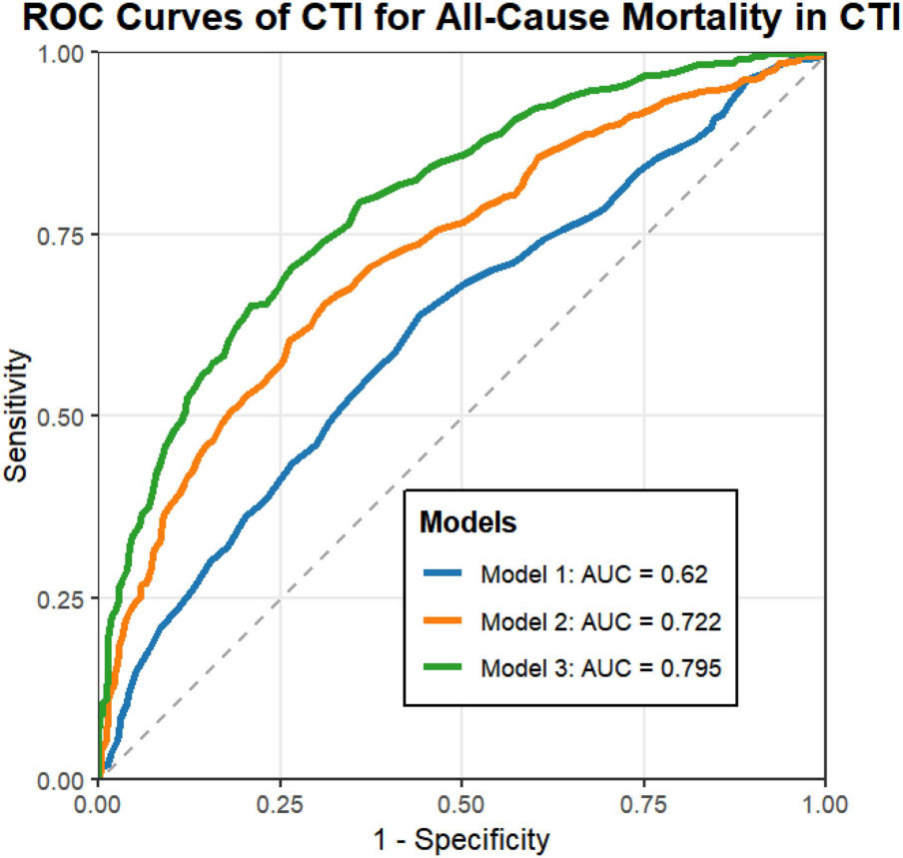
ROC curves of the three models among all CHF patients.

**Figure 6:**
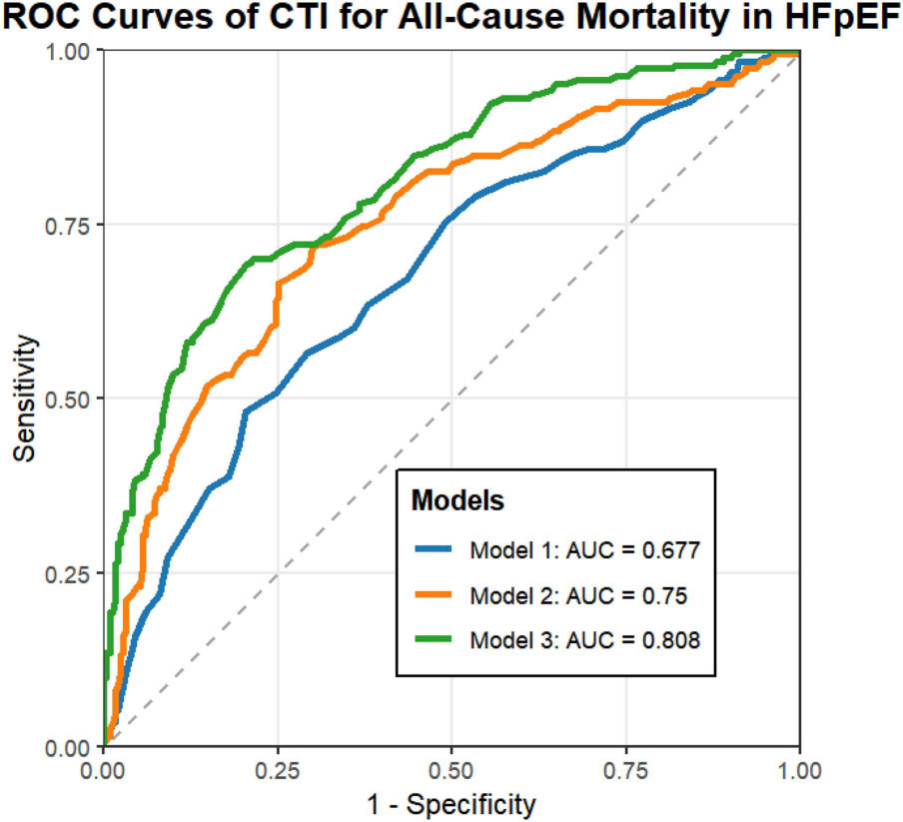
ROC curves of three models in patients with HFpEF.

**Figure 7:**
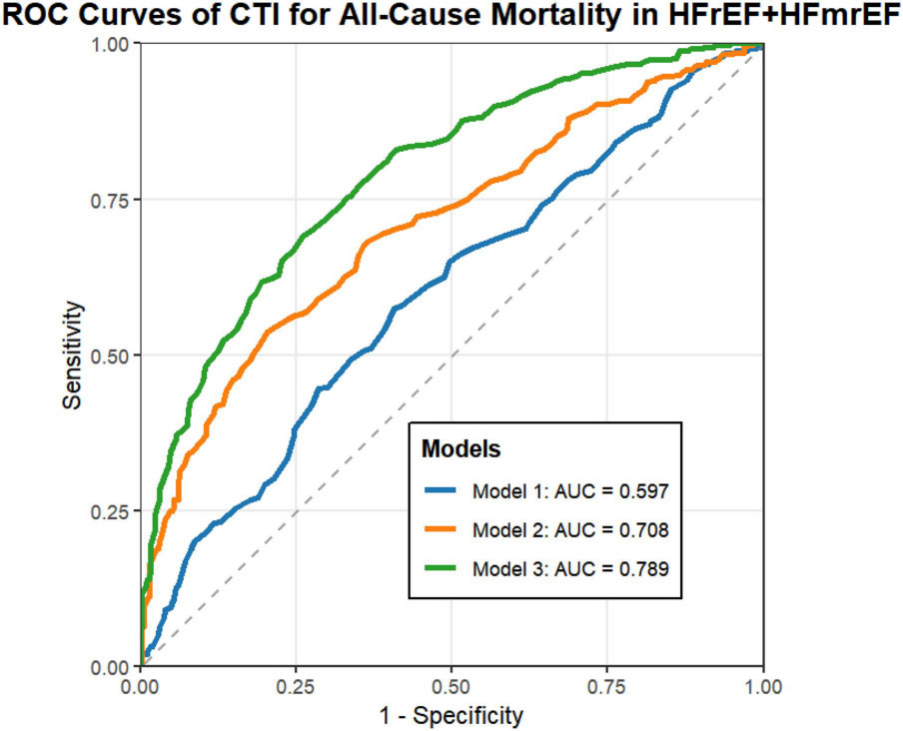
ROC curves of three models in patients with HFrEF plus HFmrEF.

In patients with chronic heart failure, Model 3 (which included CTI) showed the largest area under the ROC curve (AUC) for distinguishing patients at higher risk (Model 1: AUC = 0.620, *p* < 0.001; Model 2: AUC = 0.722, *p* < 0.001; Model 3: AUC = 0.795, *p* < 0.001) (Figure 5). We then assessed the discriminative ability of CTI across different ejection fraction subtypes. As shown in Figure 6, for heart failure patients with preserved ejection fraction (HFpEF), the AUC for Model 1 was 0.677, for Model 2 was 0.750, and that of Model 3 was 0.808 (all *p* < 0.001); Figure 7 shows that in HFrEF + HFmrEF patients, the area under the ROC curve was 0.597 for model 1, 0.708 for model 2, and 0.789 for model 3 (*p* < 0.001). These findings indicate that CTI provides good risk stratification for HFpEF and moderate discriminative value for HFrEF + HFmrEF.

**Figure 8:**
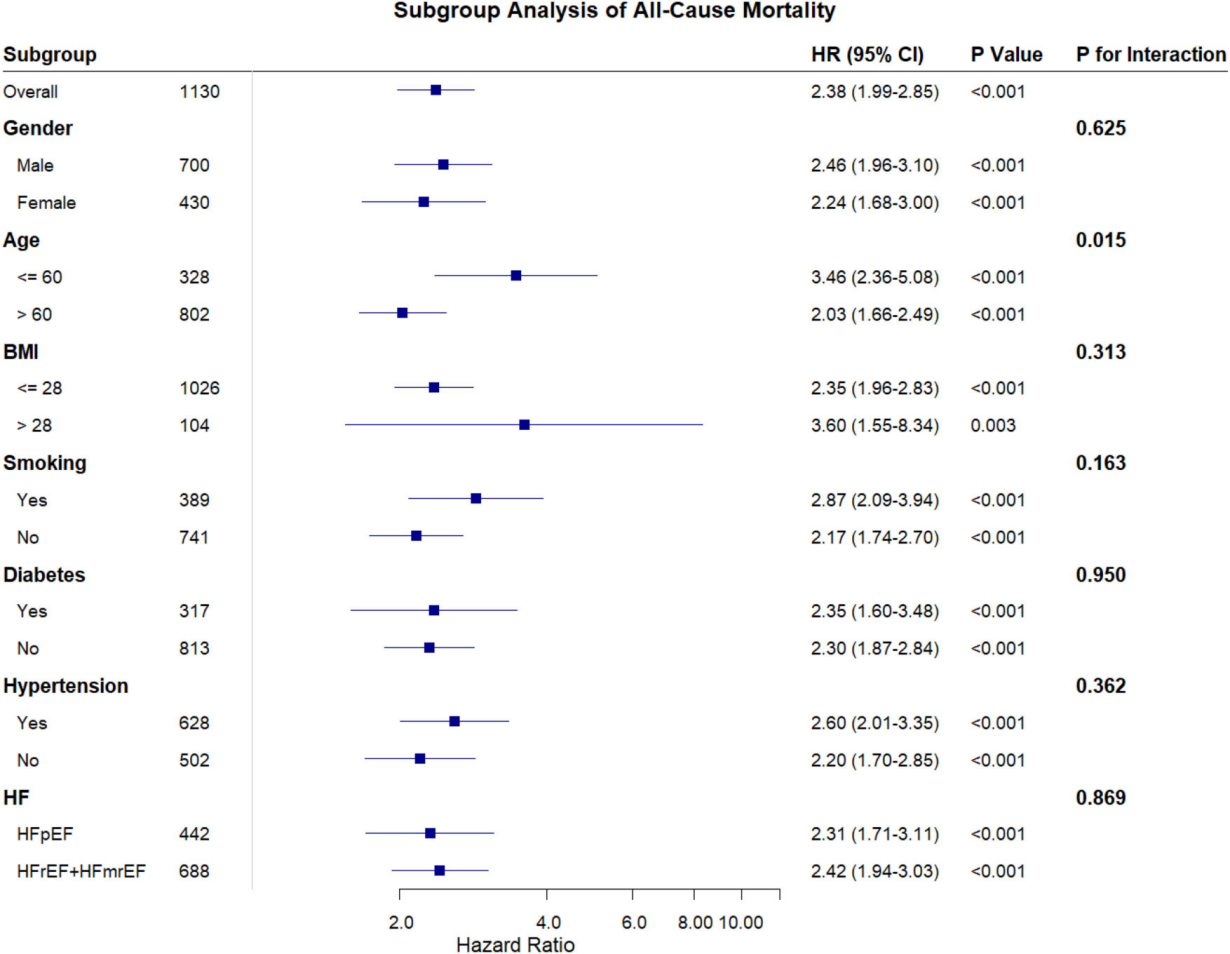
Subgroup analysis of each variable at different CTI levels.

Using the Youden index, we determined the optimal cut-off values for each model. In the overall population, Model 3 achieved an optimal cut-off of 0.2235 (sensitivity: 70.6%, specificity: 74.0%). In the HFrEF + HFmrEF subgroup, the optimal cut-off for Model 3 was 0.2244 (sensitivity: 71.7%, specificity: 71.9%). In the HFpEF subgroup, the optimal cut-off was 0.2260 (sensitivity: 67.5%, specificity: 82.1%). Detailed results for all three models across the three populations are presented in Table 4.

**Table 4:** Diagnostic performance of three models for 1-year mortality prediction in the overall population and HF subgroups.

Population	Model	Cutoff	Sensitivity	Specificity	PPV	NPV	AUC (95% CI)
Overall (*n* = 1,130, events=530)							
	Model 1	0.2517	0.64	0.558	0.561	0.637	0.620 (0.587–0.653)
	Model 2	0.2342	0.653	0.695	0.654	0.694	0.722 (0.693–0.752)
	Model 3	0.2235	0.706	0.74	0.706	0.74	0.795 (0.769–0.821)
HFrEF + HFmrEF(*n* = 688, events=339)							
	Model 1	0.2643	57.5	59.3	57.9	59	0.597 (0.555–0.639)
	Model 2	0.2808	54.3	79.4	71.9	64.1	0.708 (0.669–0.746)
	Model 3	0.2244	71.7	71.9	71.3	72.3	0.789 (0.756–0.822)
HFpEF(*n* = 442, events=191)							
	Model 1	0.2791	55	73.3	61	68.1	0.677 (0.626–0.727)
	Model 2	0.2169	72.3	69.7	64.5	76.8	0.750 (0.703–0.796)
	Model 3	0.226	67.5	82.1	74.1	76.9	0.808 (0.768–0.849)

HF, heart failure; HFrEF, heart failure with reduced ejection fraction; HFmrEF, heart failure with mildly reduced ejection fraction; HFpEF, heart failure with preserved ejection fraction; PPV, positive predictive value; NPV, negative predictive value; AUC, area under the curve; CI, confidence interval.

### Comparison of predictive value between CTI, CRP, and TyG

To directly compare CTI with CRP and TyG, a supplementary analysis was performed. The CTI model achieved a higher C-index (0.7339) than the CRP model (0.7215) and the TyG model (0.6919), along with the lowest AIC and BIC values. Likelihood ratio tests confirmed that the CTI model was superior to both the CRP model (*χ*^2^ = 37.60, *p* < 0.001) and the TyG model (*χ*^2^ = 132.78, *P* < 0.001). CTI was an independent prognostic marker of mortality (HR = 1.872, 95% CI: 1.691–2.074, *P* < 0.001)(Supplementary Table 1).

### Subgroup analysis

To assess whether the association between CTI and all-cause mortality varied across different patient subgroups, we performed subgroup analyses on age (≤60 years vs. > 60 years), sex (male/female), BMI (≤28 vs. > 28), smoking history, history of hypertension, history of diabetes, and heart failure type (HFrEF + HFmrEF vs. HFpEF). Overall, CTI was significantly associated with all-cause mortality in the entire population (HR = 2.38, 95% CI: 1.99–2.85, *p* < 0.001). The association was significant across most subgroups, including sex, BMI, smoking history, diabetes, hypertension history, and heart failure type (all *p* < 0.05), with no evidence of significant heterogeneity (all interaction *p* > 0.05). There was a significant interaction only in the age subgroup (*P* = 0.015), and the association between CTI and mortality was stronger in patients aged ≤60 years (HR = 3.46, 95%CI: 2.36–5.08, *p* < 0.001) than in those aged >60 years (Figure 8).

## Discussion

In this retrospective study, elevated CTI levels were associated with increased all-cause mortality in patients with CHF. Multivariable Cox regression identified CTI as a factor independently associated with mortality in the overall population (HR = 1.697) as well as in HFpEF (HR = 1.906) and HFrEF + HFmrEF (HR = 1.592) subgroups. In this cohort, the model incorporating CTI showed improved discriminative ability compared with models without CTI. Subgroup analyses suggested generally consistent associations across most strata, with a potential age-related interaction (stronger association in patients aged ≤60 years; interaction *p* = 0.015). Based on these findings, CTI is intended for risk stratification in patients with chronic heart failure. The RCS-derived cutoff can be used to identify patients at higher risk of all-cause mortality, which may inform decisions about follow-up intensity. Its role in clinical decision support requires prospective validation.

Insulin resistance is a hallmark of heart failure, and the TyG index is a widely used surrogate marker (6). The long-term accumulation of metabolic abnormalities—including lipid accumulation, inflammation, and oxidative stress—promotes the development of insulin resistance and contributes to heart failure progression (7). In the heart, insulin resistance impairs myocardial glucose uptake and vasodilation, disrupts lipid metabolism (leading to increased fatty acid oxidation and lipid accumulation), and promotes cardiac hypertrophy, cell death, and ventricular remodeling (8–11). At the vascular level, it induces endothelial dysfunction, characterized by impaired vasodilation, smooth muscle proliferation, and increased endothelin-1 secretion. Additionally, insulin resistance may activate the renin-angiotensin-aldosterone system (RAAS). Both pathways can ultimately promote cardiac hypertrophy and fibrosis (12–14).

In a 2010 cross-sectional study, Dinh et al. included 208 subjects who underwent selective coronary angiography. Insulin resistance was assessed using the Homeostasis Model Assessment (HOMA-IR), and echocardiographic data were measured in subjects without a history of diabetes. The study demonstrated that insulin resistance was independently associated with left ventricular diastolic dysfunction (LVDD) (15). Recent studies have shown that the association between insulin resistance and the risk of HFpEF is stronger than that of HFrEF, with substantial differences in insulin sensitivity profiles between the two HF subtypes (16). Additionally, higher insulin resistance in young adulthood has been identified as a risk factor for adverse left ventricular remodeling and dysfunction later in life (17).

Inflammation plays a key role in heart failure pathophysiology, and CRP is a widely used clinical biomarker of inflammation (18). Following myocardial injury, CRP promotes pro-inflammatory and pro-thrombotic responses and impairs vasodilation (5, 19, 20). These effects are mediated through the release of inflammatory cytokines, activation of the complement system, and endothelial dysfunction (20–22). A Framingham study (*n* = 732) investigated the relationship between serum CRP levels and the incidence of chronic heart failure (CHF) in elderly patients without a history of myocardial infarction or CHF. When the serum CRP level was greater than or equal to 5 mg/dL, the risk of chronic heart failure could increase by 2.8 times (23).

Notably, we observed a stronger association between CTI and mortality in younger patients (≤60 years). This finding is consistent with evidence from the CHARLS study, in which Zhang et al. reported that the association between CTI and cardiovascular disease was more pronounced in individuals aged 45–60 years (24). Similarly, Reinhardt et al. conducted a retrospective analysis of 2,184 patients with HFmrEF and found that non-ischemic cardiomyopathy was the most common etiology in patients under 40 years of age, whereas ischemic cardiomyopathy predominated in those aged 60–80 years (>60%) (25). This suggests that metabolic dysregulation and inflammation may play a more prominent role in younger patients with heart failure, in contrast to atherosclerosis-related mechanisms in older individuals. Finally, the CARDIA study demonstrated that cumulative exposure to insulin resistance beginning in early adulthood adversely affects left ventricular remodeling and function at middle age (17), further supporting the long-term impact of metabolic abnormalities on cardiac health in younger populations.

Our findings are consistent with previous studies linking insulin resistance and inflammation to poor prognosis in heart failure. Li et al. reported that a higher TyG index was associated with increased mortality in patients with heart failure (26), and Burger et al. demonstrated that CRP is an independent risk marker for the development of heart failure (20). However, these studies typically examined TyG and CRP separately. Recent evidence suggests that composite inflammatory indices integrating multiple biological pathways may provide enhanced prognostic value compared to single biomarkers. For example, the pan-immune-inflammation value (PIV) and HALP score are independently associated with adverse outcomes in cardiovascular diseases, particularly in acute coronary syndromes (27). Similarly, albumin-based inflammatory indices have demonstrated significant associations with hemodynamic alterations and clinical outcomes in specific cardiovascular conditions (28). These findings support the broader concept that combining inflammatory and metabolic parameters—as we have done with the CTI integrating CRP and TyG—may improve risk stratification in heart failure patients. Our study extends the existing literature by showing that combining these two markers into a single metric (CTI) provides incremental prognostic value over either component alone, as indicated by lower AIC/BIC values and a higher C-index for the CTI model compared with models containing only TyG or CRP.

The C-reactive protein-triglyceride glucose index (CTI), in conjunction with C-reactive protein (CRP) and TyG index, serves as a diagnostic tool for heart failure by assessing inflammation and insulin resistance. In earlier research, CTI has been posited as a potential clinical indicator for predicting cancer and depression (29, 30). In the most recent research, published in 2025, Sun et al. have confirmed that CTI is significantly associated with the incidence and mortality of CVD. Furthermore, CTI can serve as an independent predictor of heart failure, coronary heart disease, stroke, angina pectoris, and cardiac arrest (31). However, there are currently no studies that have further analyzed the impact of CTI on the prognosis of patients with heart failure of different ejection fractions. For the first time, we explored the predictive value of CTI for all-cause mortality in CHF patients with different ejection fractions.

We acknowledge that HFmrEF is increasingly recognized as a distinct but heterogeneous phenotype within the heart failure spectrum (32). Nevertheless, several considerations support combining HFrEF and HFmrEF in our analysis. First, Shah et al. reported that HFpEF is the most common subtype in Asian populations (52.2%) (33, 34). Given that HFpEF represents the predominant phenotype in this region, it is methodologically appropriate to treat the remaining patients (HFrEF and HFmrEF combined) as a single comparator group. In our cohort, HFmrEF alone accounted for only 219 patients; combining HFmrEF with HFrEF increased the sample size of the non-HFpEF group to 688 (compared with 442 for HFpEF), thereby enhancing statistical power for subgroup analyses and multivariable modeling. Furthermore, previous studies have demonstrated that HFmrEF and HFrEF have comparable mortality risk, whereas both differ significantly from HFpEF (35). The nationwide Korean KorHF III registry reported that after inverse probability weighting, HFmrEF and HFrEF showed no significant difference in the composite outcome of all-cause death or heart transplantation (36). These findings indicate that HFmrEF and HFrEF form a clinically similar prognostic group distinct from HFpEF, further supporting their combined analysis.

Several limitations should be acknowledged. First, the single-center retrospective design of this study inherently limits causal inference and introduces potential selection bias. Retrospective data collection may be subject to information bias due to incomplete or inconsistent medical records, and the lack of standardized protocols for CTI measurement across different centers limits the generalizability of our findings. Moreover, our single-center setting reflects the clinical practices and patient referral patterns of a tertiary academic hospital, which may not be representative of community-based or lower-volume centers. Second, despite adjusting for multiple covariates in the multivariable Cox regression models, the possibility of residual confounding cannot be excluded due to unmeasured factors such as medication adherence, frailty, nutritional status, or changes in guideline-directed medical therapy over time. Third, the relatively small sample size—particularly after stratification into ejection fraction subtypes—may have reduced statistical power for certain subgroup analyses. Fourth, this study only used CRP, TG, and FBG levels measured at admission, without longitudinal monitoring of these parameters during follow-up; therefore, the prognostic impact of dynamic changes in CTI remains unknown. Fifth, the study population predominantly consisted of patients with NYHA class III–IV symptoms and elevated BNP levels (≥500 pg/mL), reflecting relatively advanced heart failure. While this represents a clinically important high-risk subgroup in whom prognostic tools are most needed, it also limits the generalizability. Our findings are most directly applicable to patients with moderate-to-severe heart failure and should not be freely extrapolated to patients with milder symptoms (NYHA I-II), those with lower BNP levels, or community-based heart failure populations with stable disease. Sixth, diabetes mellitus was not included in our primary multivariable Cox models despite its significant univariate association (*p* < 0.001). However, a *post-hoc* sensitivity analysis adding diabetes yielded nearly identical HRs for CTI (all changes <5%; Supplementary Table 2), and the interaction was non-significant (*p* = 0.95). Nevertheless, this omission remains a limitation. Seventh, this study focused on model discrimination rather than calibration, as the primary aim was to evaluate the prognostic role of CTI rather than to develop a clinical prediction model. Calibration assessment should be considered in future validation studies. Given these limitations, particularly the retrospective design and potential residual confounding, our findings should be interpreted as hypothesis-generating rather than conclusive.

Prospective multi-center studies with larger cohorts, longitudinal CTI measurements, and external validation in independent populations are warranted to further validate the prognostic value of CTI in patients with different heart failure subtypes.

## Conclusions

CTI is an independent prognostic marker for all-cause mortality in CHF patients across different ejection fraction subtypes. Higher CTI levels are associated with increased mortality risk, with a stronger association observed in HFpEF compared with HFrEF + HFmrEF.

## Data Availability

The datasets presented in this article are not readily available because the data analyzed in this study contains sensitive information (such as patient records, geographical locations, etc.), even after anonymization, there is still a risk of re-identification. Currently, the original data cannot be made public. However, this study has provided a detailed data processing procedure in the paper to ensure that the results are completely reproducible. If there are any further requirements, we will cooperate actively. Requests to access the datasets should be directed to ydyyclx@163.com.
