# Supplementary material for: The influence of C-reactive protein-triglyceride-glucose index (CTI) on the prognosis of heart failure patients with different ejection fractions
Source: Front Cardiovasc Med. 2026 Jun 8;13:1808481. doi: 10.3389/fcvm.2026.1808481 (PMC13284143; doi:10.3389/fcvm.2026.1808481)
Supplement: Supplementary file 1 [file Table1.docx]

Supplementary Table 1: Comparison of Cox regression models for predicting mortality.

| Variables | Model A (CRP) | Model B (TyG) | Model C (CTI) |
| --- | --- | --- | --- |
| HR (95% CI) | 1.011 (1.009-1.013) | 1.262 (1.104-1.441) | 1.872 (1.691-2.074) |
| P-value | <0.001 | <0.001 | <0.001 |
| C-index | 0.7215 | 0.6919 | 0.7339 |
| AIC | 6725.87 | 6821.05 | 6692.27 |
| BIC | 6755.78 | 6850.96 | 6722.18 |
| Likelihood ratio χ² | 37.60 | 132.78 | Reference |
| P-value for comparison | <0.001 | <0.001 |  |
| All models were adjusted for Age, Gender, lgBNP, RBC, Sodium, and GFR. HR = hazard ratio; CI = confidence interval; AIC = Akaike information criterion; BIC = Bayesian information criterion. | | | |

Supplementary Table 2 Sensitivity analysis: Primary multivariable Cox model for CTI with additional adjustment for diabetes mellitus.

| Subgroup | Primary multivariable Cox model HR (95% CI) | Multivariable Cox Model + diabetes HR (95% CI) |
| --- | --- | --- |
| Overall cohort (n = 1130) | 1.697 (1.522–1.891) | 1.740 (1.552–1.950) |
| HFrEF+HFmrEF (n = 688) | 1.592 (1.389–1.824) | 1.624 (1.407–1.874) |
| HFpEF (n = 442) | 1.906 (1.565–2.321) | 1.967 (1.592–2.431) |
| All models were adjusted for the same covariates as the primary multivariable Cox models, plus diabetes mellitus. | | |

Supplementary Table 3: Assessment of overfitting risk for the multivariable Cox regression model.

| Subgroup | N | Events | Covariates | EPV | Apparent C-index | Bootstrap-corrected C-index (95% CI) | Optimism |
| --- | --- | --- | --- | --- | --- | --- | --- |
| Overall | 1130 | 530 | 18 | 29.4 | — | — | — |
| HFrEF+HFmrEF | 688 | 339 | 18 | 18.8 | — | — | — |
| HFpEF | 442 | 191 | 18 | 10.6 | 0.8 | 0.809 (0.774-0.838) | -0.008 |
| EPV: events per variable; CI: confidence interval. | | | | | | | |
